# Prediction of borderline personality disorder based on childhood trauma with the mediating role of experiential avoidance

**DOI:** 10.3389/fpsyt.2024.1382012

**Published:** 2024-11-06

**Authors:** Boliang Jiang

**Affiliations:** Management Innovation and Evaluation Research Center, School of Management, Tianjin University of Commerce, Tianjin, China

**Keywords:** borderline personality disorder, childhood trauma, experiential avoidance, statistical approaches, questionnaire

## Abstract

**Introduction:**

Traits of borderline personality disorder are important for the determination of the prognosis of mental illnesses and in evaluating risks of negativity as well as impulsivity. But, there is a lack of information about the distribution characteristics of borderline personality disorder traits and symptoms within clinical groups. The goal of the current study was to predict borderline personality disorder based on childhood trauma, using experiential avoidance as a mediator.

**Methods:**

All male patients hospitalized in local psychiatric health centers with a diagnosis of borderline personality disorder comprised the statistical population of the current study. The number of 60 patients were selected by the purposeful sampling method. The questionnaire included the Childhood Trauma Questionnaire (CTQ), the Experiential Avoidance Questionnaire (AAQ-II), and the Borderline Personality Disorder Symptoms (BSL-23).

**Results and discussion:**

The results demonstrated that there is a considerable and positive relationship between childhood trauma and experiential avoidance (r = 0.711, p< 0.01). In the mediating model, childhood trauma had significant direct predictive effects on borderline personality disorder (β = 0.546, p< 0.01). Also, between childhood trauma and BPD, experienced avoidance acts as a moderating factor. (β = 0.304, p< 0.01).

## 1 Introduction

Borderline personality disorder (BPD) affects approximately 1% of the general, about 10% of the outpatient psychiatric, and approximately 20% of the inpatient psychiatric populations (1). It is related to poor professional, functional, and social behaviors, leading to a decrease in quality of life generally (2). In the Diagnostic and Statistical Manual of Mental Disorders (DSM-5), the disorder of Borderline personality disorder is given a definition of “A pervasive tendency toward instability of interpersonal relationships, self-image, and impacts, and significant impulsivity commencing in early adulthood and present in a range of contexts” (3). Based on this, the main symptomatic dimensions of borderline personality disorder are impulsivity, emotion regulation, disturbance in identity, and interpersonal problems.

These diseases start to show up in adolescence and continue for the rest of a person’s life (4). It is a severe clinical disease with negative effects on many levels. Although childhood trauma is quite common, it is still unknown how this trauma affects how BPD symptoms develop (5–7). These behaviors are the result of issues with early childhood parenting and the relationship between parents and children. These individuals typically experience verbal abuse, neglect, inattention, or poor training (8).Occasionally a history of divorce, premature deaths of parents, and traumas in the childhood of people with this disorder can also be seen (9).

Developmentally speaking, insecure attachment related to childhood trauma is linked to borderline personality disorder (10). Therefore, one of the fundamental elements that has a role in people’s tendency to have BPD is childhood trauma (11).

A model that has been able to explain the causes of borderline personality disorder to a great extent is the model of Zanarini (12). According to this model, borderline personality disorder is created as a result of a complicated mix of three factors: a person’s temperament, childhood traumas and challenges, and relatively subtle neurological and biochemical distortion results (13, 14). In line with these theories, many studies have been conducted, as a result of which borderline personality disorder is created from an unstable and unreliable childhood life environment and is specifically characterized by emotional misbehavior and negative emotions (15, 16).

The definition of childhood trauma includes physical and emotional neglect and physical as well as sexual abuse of people under the age of 18 by someone in a protective role. Childhood trauma can cause a profound and long-lasting influence. Children who have suffered severe psychological injuries imagine the world to be a terrifying and scary place. Childhood trauma that is not healed, this basic concept of helplessness as well as fear carries over into adulthood and creates the condition for more trauma (5). The findings of many works have shown that childhood traumas play a vital role in the occurrence of personality disorders, especially borderline personality disorder (5, 17–20). There is a close relationship between dissociation and childhood trauma in borderline personality disorder. It was shown that dissociation plays a vital role in the activity of areas of the brain that are responsible for processing unpleasant information (related to trauma) and also has destructive effects on working memory in people with borderline personality disorder. It was suggested that borderline personality disorder is more related to flashbacks about past experiences. Also, more studies have shown the relationship between childhood traumas and the tendency to develop borderline personality disorder (16, 21, 22).

Also, experiential avoidance (EA) has been proposed as one of the mediating variables related to childhood traumas and its mediating role in determining borderline personality disorder. The present work aims to evaluate in more detail the effect of experiential avoidance on borderline personality disorder. Experiential avoidance is the innate human desire to prevent threatening or uncomfortable situations. Also, experimental avoidance is an unwillingness to feeling depressed or the perceived need to control (23).

Experiential avoidance consists of two components: Refusal to engage with personal experiences such as physical sensations, emotions, and memories, as well as behavioral contexts, and attempting to prevent painful experiences or circumstances that lead to these experiences being recalled (24–26). The performance of experiential avoidance handles or minimizes the effect of disturbing experiences and creates short-term relaxation, which, as a result of this action, affects the behavior in a negative way. This avoidance becomes problematic when it interferes with the person’s daily activities (27). A person’s constant and continuous desire to experience uncomfortable thoughts and feelings and avoid related experiences is related to a wide range of problems. Experiential avoidance is also related to suicidal thoughts, deliberate self-harm, and factors that can provide the basis for self-harm behaviors, including emotional disorders (28, 29).

People with BPD typically struggle to accept their unpleasant feelings, and this inability to employ effective techniques for regulating emotions causes and exacerbates additional pathological symptoms in these individuals (30). In these individuals, dyslexia and distress tolerance may be related to a lack of emotion control (31).

So far, the research studies performed in this scope have mainly been focused on the normal population or other kinds of illnesses. There are little studies about BPD patients in this regards. Also, previous works have not considered the mediating factor of experiential avoidance. So, in this work, in order to fill the gap, the impact of experiential avoidance on BPD was investigated. So, taking into account the high incidence and detrimental impacts of BPDs on the individual and the community, it will be highly important to identify the factors and predict the variables that have a significant influence on this disorder. The results of this research study will provide a proper answer to this question. Does childhood trauma have a role in predicting BPD with the mediating role of experiential avoidance?

## 2 Methods

### 2.1 Participants

The current work is a description that correlates its primary design with its intended use. There were sixty male patients with BPD in the statistical population. The male patients were selected because the number of male patients with this disorder was more available to conduct the study. Also, focusing on men with borderline personality disorder has allowed us to address specific aspects of the disorder in this group that have not been investigated in previous studies with more diverse samples. They were chosen from the community of people who were met the criteria for having BPD by a psychiatrist (diagnosis sheet, or according to diagnostic criteria of DSM-5 and interviews such as SCID-5. The following criteria were considered:

1. Male with 18-40 year olds
2. Being able to read and write
3. Diagnosis of disorders based on DSM-5 and SCID-5 criteria

Patients with psychotic and bipolar symptoms, suicidal thoughts, and physical illnesses were excluded from the current research study.

### 2.2 Childhood trauma questionnaire

The childhood trauma questionnaire (CTQ) was designed by Bernstein et al. (32, 33) to measure childhood trauma. It evaluates five types of abuse, including sexual, emotional, and physical abuse, and physical/emotional neglect. It contains twenty-eight questions; 25 questions are applied to measure the primary elements of the questionnaire, and three questions are applied to diagnose people who deny their childhood issues. The Likert scale, which ranges from never to always, is used to score items. Cronbach’s alpha coefficients for emotional, sexual, and physical abuse, and emotional/physical neglect, are equivalent to 0.95, 0.87, 0.86, 0.89, and 0.78, respectively. The ratings of therapists were found to be between 0.59 and 0.78 (33). Brodsky et al. (34) and Roy (35) reported the reliability of this tool using two test-retest methods and Cronbach’s alpha in the range of 0.79-0.94.

### 2.3 Acceptance and action questionnaire

This tool was developed by Bond et al. (36) and is the most widely used measure of experiential avoidance and psychological inflexibility. It has seven items. It is graded based on a 7-point Likert scale (from never = 1 to always = 7). A higher score indicates greater experiential avoidance as well as inflexibility. Also, the value of Cronbach’s alpha reliability for the whole test is 0.71, and the retest reliability is 0.81 (36).

### 2.4 Borderline symptom list (BSL-23) for BPD

This scale is a 23-item self-report form developed by Bohus et al. (37) to diagnose BPD. This scale is sensitive to changes in treatment length. Cronbach’s alpha coefficient was also reported between 0.49 and 0.77. Data analysis was also performed using path analysis tests and structural equations by PLS software.

## 3 Results

The total sample size included 60 patients with borderline personality disorder. The age of the people was the most frequent in the age range of 20-30 years [(30 people) (50%)], and the lowest age range was in the age range of under 20 years (5, 8.4%) people. 25 (41.6%) people were in the age range of 31-40. The educational status of the sample includes 30 people (50%) with diploma and post-graduate degrees; 22 people (36/7%) were under diploma; 3 (5%) people had bachelor’s; and 5 people (8.3%) had a master’s degree or higher, respectively. 37 people (61.7%) from the sample group were single, 20 (33.3%) were married, and 3 (5%) were divorced. The frequency of employment distribution among the respondents includes 41 (68.4%) unemployed-retired people, 11 (18.3%) free-lance people, and 8 (13%) employed people. **Table 1** shows the descriptive indices of the studied variables.

**Table 1:** Descriptive indices of variables.

Variables	Number of items	Average	Standard deviation	Minimum	Maximum
Childhood trauma	25	3.436	0.568	1.50	4.64
Experiential avoidance	7	3.481	1.169	1.14	6.43
Borderline personality disorder	23	3.330	0.668	1.57	4.65

The skewness and kurtosis values were used to determine the normality of the data. An absolute skewness amount ≤2 or an absolute kurtosis value ≤4 can be considered as reference values for the calculation of significant normality. The results are given in **Table 2**. Skewness, and kurtosis coefficient, and standard error for all variables are between -2 and 2, which means the distribution of data is normal.

**Table 2:** Normality of variables in the current study.

Variables	Skewness coefficient		Kurtosis coefficient		Test of normality
	Statistic value	Standard error	Statistic value	Standard error	
Childhood trauma	-0.933	0.309	1.575	0.608	It is Normal.
Experiential avoidance	0.258	0.309	-0.063	0.608	It is Normal.
Borderline personality disorder	-0.208	0.309	0.017	0.608	It is Normal.

**Figure 1:**
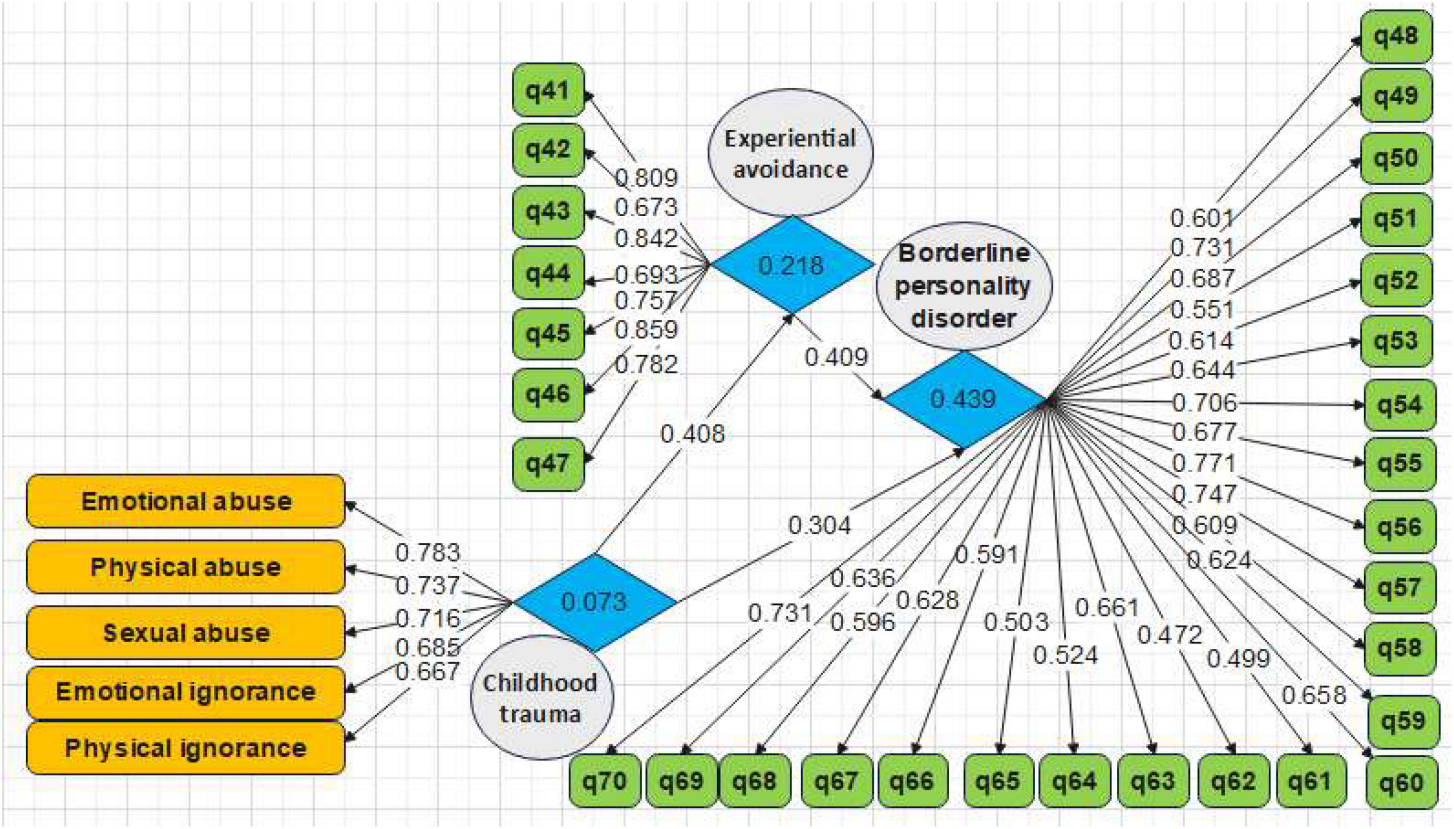
Research model with standardized factor loading coefficients (evaluation of measurement models, Model 1).

**Figure 2:**
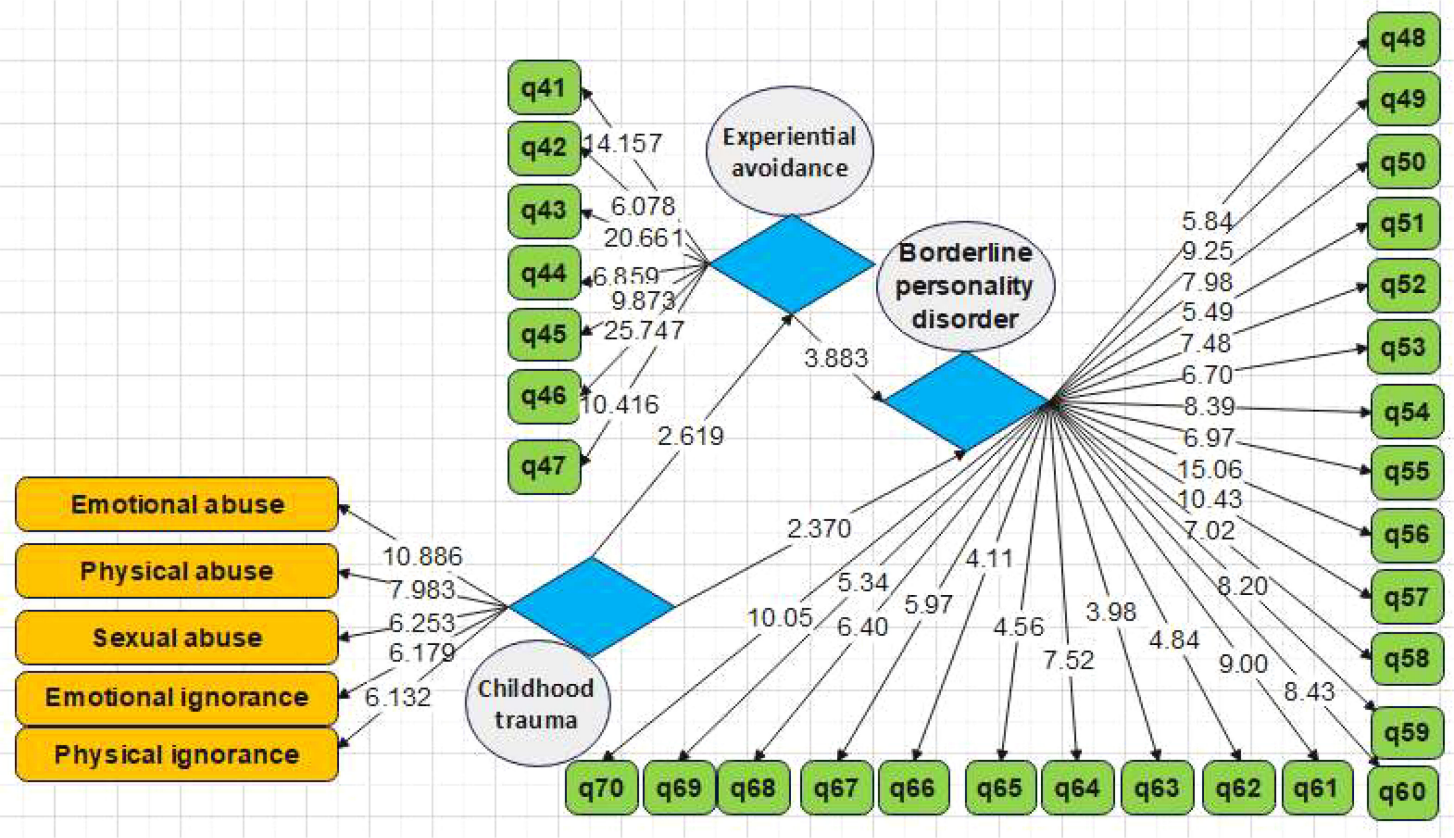
Research model based on t values (evaluation of measurement models, Model 2).

In order to assess the measurement models, the criteria of the reliability analysis test, including Cronbach’s alpha as well as composite reliability coefficient, convergent validity such as significant factor loadings, average variance extracted (AVE), homogeneity, and comparing CR with AVE, and convergent validity for Fornell’s and Larcker’s test (38) were investigated. According to the first and second models provided in **Figures 1**, **2**, the values of factor loadings and significant coefficients of all items were calculated to be more than 0.4 and 1.96, respectively. Therefore, it is inferred that the convergent validity of the variables in the model has been validated. **Table 3** shows Cronbach’s alpha as well as the composite reliability coefficients of the developed model. It can be seen that both parameters are in the acceptance range.

**Table 3:** Cronbach’s alpha as well as composite reliability coefficient results.

Variables	Cronbach’s Alpha>0.7	Composite Reliability>0.7
Childhood trauma	0.765	0.842
Experiential avoidance	0.889	0.913
Borderline personality disorder	0.932	0.939

**Table 4** presents the convergent validity using the average variance extracted (AVE) method. The appropriate value for AVE is 0.5 (38). In **Table 4**, all results are greater than 0.5, which means that the results are in the acceptance range based on this index. Furthermore, CR is higher than AVE in all variables. Therefore, the model is valid in terms of convergent validity.

**Table 4:** Assessment of the convergent validity using the average variance extracted (AVE) method.

Variables	AVE	CR	CR>AVE
Childhood trauma	517	842	OK
Experiential avoidance	602	913	OK
Borderline personality disorder	505	939	OK

Divergent validity was studied using the Fornell-Larcker method (38) and the results are shown in **Table 5**. The findings showed that the quantitative root of the AVE of variables is higher than the correlation values between them. It means that the dependent variables have more interaction with the questions than other variables.

**Table 5:** Divergent validity results based on the Fornell-Larcker method.

Variables	Childhood trauma	Experiential avoidance	Borderline personality disorder
Childhood trauma	-	-	-
Experiential avoidance	0.447	-	-
Borderline personality disorder	0.524	0.579	-

After analyzing the measurement model, structural models were evaluated. Firstly, the t value was used to investigate structural models. **Table 6** shows the standardized loading coefficients and t value between the variables. According to **Table 6**, the absolute value of the t value between independent and dependent variables is higher than 1.96, and they are significant at the 95% level. In other words, it shows the appropriateness of the structural model.

**Table 6:** Standardized loading coefficients and t values between the variables http://www.statmodel.com/discussion/messages/9/276.html?1531184421.

Independent variables	dependent variable	Path coefficient (β)	t value
Childhood trauma	Experiential avoidance	0.408	2.768
Childhood trauma	borderline personality disorder	0.304	2.339
Experiential avoidance	borderline personality disorder	0.409	3.709

A statistical hypothesis test called the goodness-of-fit (GOF) evaluates how well observed and predicted data match each other. Goodness-of-fit is a measure of how close the data “agrees” with your model.

The GOF was determined using communality and R^2^ values.


(1) $$GOF=\sqrt{\bar{Communalities}\times \bar{R^{2}}}=\sqrt{0.294\times 0.243}=0.267$$

The communality and R^2^ values were obtained to be 0.294 and 0.243, respectively. The GOF was calculated, and it was 0.267. In the GOF method, 0.01, 0.25, and 0.36 values are defined as weak, moderate and strong (39). As the calculated value is between 0.25 and 0.36, it means that the model is strongly fit, and the overall fitness of the model is thus confirmed.

Pearson’s correlation test was also used to study the relationship between childhood trauma and experiential avoidance. The results are shown in **Table 7**. There is a significant positive correlation of 0.71 between childhood trauma and experiential avoidance, based on a significance level of less than 0.05 and correlation coefficients. In other words, with increasing childhood trauma, experiential avoidance will also increase, and vice versa. The role of childhood trauma in the prediction of borderline personality disorder was evaluated, and the results are provided in **Table 8**. It was observed that the t value is 2.339, which is higher than 1.96. It means that the effect of childhood trauma on borderline personality disorder, with a path coefficient of 0.235 and a probability of 95%, is significant. The positive path coefficient (β = 0.304) between the variables implies that, as childhood trauma increases, borderline personality disorder will also increase. Therefore, the role of childhood trauma in borderline personality disorder was confirmed.

**Table 7:** Pearson correlation coefficient between variables.

Variables	Experiential avoidance	
Childhood trauma	Correlation value	0.711
	P value	0.001

**Table 8:** Evaluation of the role of childhood trauma in predicting borderline personality disorder.

Statistical Hypothesis	Path Coefficient	T-value	Direction of relationship	Result
Childhood trauma→ borderline personality disorder	0.304	2.339	Positive and straight	Confirmed

Furthermore, the role of the experiential-avoidance mediator in the relationship between childhood trauma and borderline personality disorder was investigated. **Table 9** shows the effect of childhood trauma on BPD according to the mediating role of experiential avoidance. In the first stage and primary model, the effect of childhood trauma alone is estimated to be significant, with a path coefficient of 0.546. In the next stage, experiential avoidance was included in the model and was used between the two variables of childhood trauma and borderline personality disorder. The path coefficient between two main variables was reduced from 0.546 to 0.304, and it is still significant. Therefore, the mediation of experiential avoidance in the relationship between childhood trauma and borderline personality disorder is confirmed with a probability of 95%.

**Table 9:** The results of investigating the effect of childhood trauma on borderline personality disorder according to the mediating role of experiential avoidance.

Baron-Kenny test items	Path	Path Coefficient	significant	p-value
Without the intervention of the mediator variable and modeling separately (models 5 and 6 provided in **Figures 3**, **4**)	Childhood trauma→ borderline personality disorder	0.546	7.06	0.001
Entering the mediator variable and modeling with all variables at once (models 1 and 2)	Childhood trauma→ Experiential avoidance	0.408	2.678	0.008
	Experiential avoidance → borderline personality disorder	0.407	3.709	0.001

**Figure 3:**
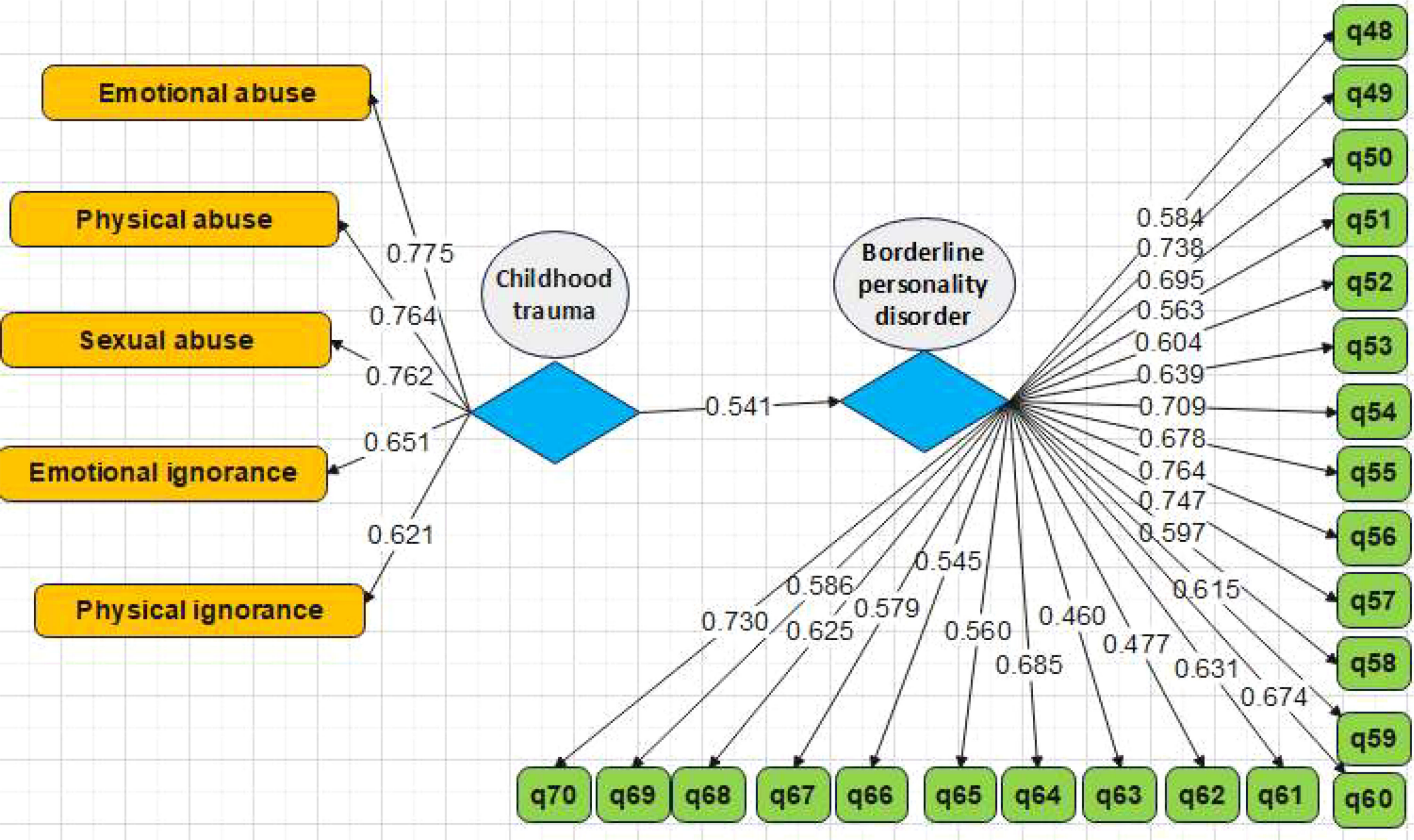
Conceptual model for investigation of the effect of childhood trauma on borderline personality disorder based on standardized coefficients (without intervening mediator variable).

**Figure 4:**
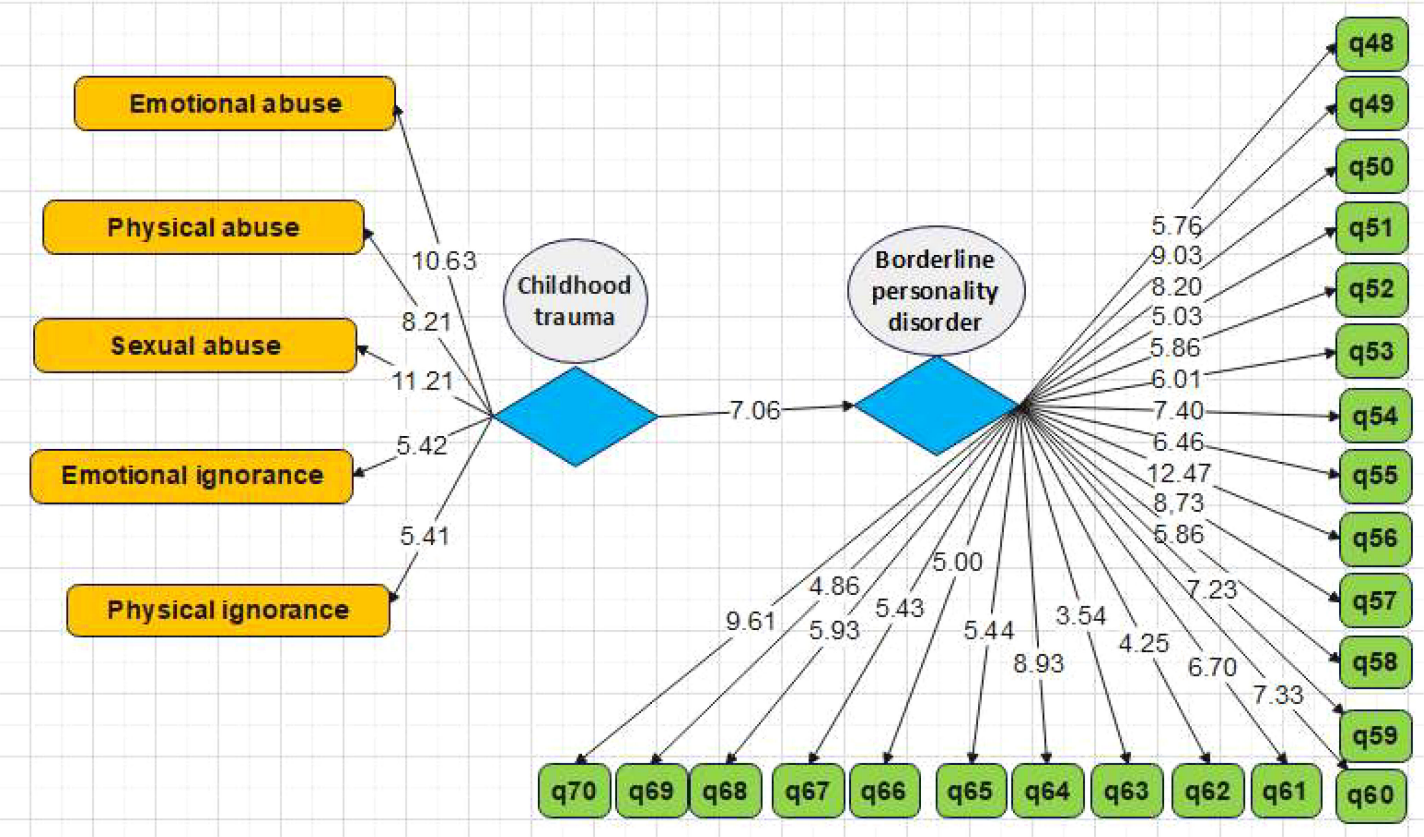
Conceptual model for investigation of the effect of childhood trauma on borderline personality disorder based on t value (without intervening mediator variable).

## 4 Discussion

The current work assessed tolerance-distress and childhood trauma in prediction of BPD with experiential avoidance as the mediating role. The results of the present study showed that there is a considerable and positive correlation between childhood trauma and experiential avoidance. The results of the present study are consistent with the results of previous works (8, 9, 16, 40–44).

In explaining the results, it can be stated that according to Bowlby’s attachment theory, the formation of a safe attachment acts as a safe anchor against psychological trauma and provides successful emotional growth. For this reason, the experience of difficult and harmful events, especially the experiences that were shaped by the caregiver, has a determining effect on the intellectual, emotional, and behavioral systems of a person towards themselves and others, and in this way, it can lead to the formation of positive and negative metacognitive opinions (45).

A child who has been abused or mistreated develops positive beliefs about worrying and re-examining threatening cases in order to prevent further possible abuse and mistreatment (46).

Repeated experiences of worry and its consequences lead to the formation of meta-anxiety or negative meta-cognitive beliefs about worry, such as the uncontrollability of thoughts. According to the perspective of metacognition, in order to deal with meta-anxiety, a person resorts to negative emotion regulation strategies such as rumination, threat monitoring, thought control, thought suppression, and avoidance, which lead to personal processing focused on the threat, so that anxiety and the continued feeling of danger do not allow cognition to return to a normal and threat-free environment (47).

In this study, experiential avoidance was specifically focused on as a coping strategy. When experiential avoidance is used as a long-term strategy, it limits healthy experiences of psychological phenomena and prevents a person from accessing internal and important information. People who have a history of injury or trauma are more likely to develop borderline personality disorder after a traumatic event.

Also, the results of the current work demonstrated that childhood trauma plays a considerable role in prediction of BPD. The results of the present study were in line with the findings of previous studies (16, 21, 48). In explaining the results, it can be stated that the model that has been able to explain the causes of borderline personality disorder to a great extent is the Zanarini model (13). According to this model, the formation of borderline personality disorder results from a complex combination of three components: the individual’s temperament, childhood traumas and challenges, and relatively subtle neurological and biochemical distortions (13). In line with these theories, borderline personality disorder arises from an unstable and unreliable childhood life environment and is specifically characterized by emotional misbehavior and negative emotions (16, 49). Although all children begin their lives with absolute dependence on caregivers, children must eventually become independent. Based on this, Masterson (50) believes that in the family of borderline patients, the tendency of the mother to maintain a close relationship with the child in parallel with the gradual growth of autonomy in the child is challenging. According to Masterson’s belief, the mother, not the patient, is ultimately responsible for the creation of borderline personality disorder. According to Masterson (50), because the primary caregivers of patients with borderline personality disorder often have great fears of rejection, they are likely to use their children as transference subjects, which gives them a sense of security (51), which leads to a kind of relationship in which the roles are reversed. Such relationships are often observed between borderline patients and their parents. People with borderline personality disorder react to repeated threats of abandonment by the primary caregiver and become vulnerable to depression resulting from abandonment. This indicates their belief that their existence depends on the existence of others who satisfy their needs and maintain their lives (28).

And finally, the results of the current work demonstrated that experiential avoidance plays a considerable role in the relationship between childhood trauma and predicting borderline personality disorder. The results are consistent with previous studies (30, 52). In the explanation of the results, it can be stated that experimental avoidance is related to suicidal thoughts, committing suicidal behavior, unintentional self-harm behavior without suicidal purpose, intentional self-harm, and the factors that can provide background for self-harm behaviors, including emotional disorders and perceived stress (53).

As a result, it can be said that validation leads to changes in emotional beliefs, and in this way, even changes the emotion itself. A common strategy to avoid discomfort is to hide emotional responses. This situation can also affect physiological arousal and possibly increase it. By hiding the discomfort, others are denied the possibility of empathizing with our emotions or speaking for us about their similar experiences and struggles. External validation is one of the methods for strengthening and increasing self-compassion. Finally, experiential avoidance often leads to behavioral avoidance or engagement in behaviors that interfere with a person’s functioning. In addition to the more obvious effects such as drug use, binge eating, or self-harm, experiential avoidance can have subtle but important consequences for one’s relationships in everyday life. In this way, it prevents people from fully engaging with their relationships and pursuing career paths, goals, values, and standards that are important to them. Furthermore, it effectively prevents them from dealing with the stressful conditions of their lives.

## 5 Conclusion

The results of this work indicated that there is a relationship between childhood trauma and experiential avoidance. Childhood trauma also plays a considerable role in prediction of BPD. Experiential avoidance also plays a significant role in the relationship between childhood trauma and BPD. In fact, when experiential avoidance is used as a long-term strategy, it limits healthy experiences of psychological phenomena and prevents a person from accessing internal and important information. There is a greater possibility of getting borderline personality disorder for people with a history of injury or trauma.

The present study had a number of limitations, like a non-random sampling method, self-reporting tools, and a small sample size. It was suggested that the developed method in the current study can be implemented on other age as well as non-clinical groups in future works. The using of random sampling methods or shorter questionnaires can improve the results obtained from participants. Furthermore, the experts in this field should focus on the aspect of childhood trauma when dealing with borderline personality disorder patients.

## Data Availability

The original contributions presented in the study are included in the article/supplementary material. Further inquiries can be directed to the corresponding author.
